# Do herbivorous fish amplify the competitive dominance of floating-leaved plants over submerged plants?

**DOI:** 10.1098/rsbl.2026.0343

**Published:** 2026-10-28

**Authors:** Manli Xia, Zhong-Ming Ye, Guoqing Quan, Wei Liu, Hui Ma, Wei Li, Yu Cao

**Affiliations:** ^1^ Research Center of Aquatic Plant and Watershed Ecology, Wuhan Botanical Garden, Chinese Academy of Sciences, Wuhan, Hubei, People's Republic of China; ^2^ College of Horticulture and Gardening, Yangtze University, Jingzhou, Hubei, People's Republic of China; ^3^ College of Resources and Environment, Huazhong Agricultural University, Wuhan, People's Republic of China; ^4^ Yani Wetland Ecosystem Positioning Observation and Research Station, Xizang University, Lhasa, Tibet, People's Republic of China; ^5^ Key Laboratory of Biodiversity Conservation and Ecological Restoration, Shannan, People's Republic of China; ^6^ Nyingchi National Botanical Garden, Nyingchi, People's Republic of China

**Keywords:** asymmetric competition, biotic interactions, community succession, selective herbivory

## Abstract

Growing interest in the relationship between herbivorous fish and aquatic plants has revealed multiple pathways by which fish can influence aquatic plant biomass and coverage. However, the impact of herbivorous fish on species competition and community assembly in aquatic plants remains poorly understood. Here, based on a mesocosm experiment with four aquatic plant species (two floating-leaved and two submerged) and one herbivorous fish, we quantified competition coefficients among plant species and fish effects using a modified Ricker model. Floating-leaved plants exhibited stronger intraspecific than interspecific competition, indicating competitive superiority over submerged species. Crucially, selective grazing by herbivorous fish on submerged plants indirectly facilitated the dominance of floating-leaved species, exacerbating competitive asymmetry. These results demonstrate that herbivory indirectly affects aquatic plant community structure, not simply by removing biomass but also via higher-order interactions. Our research extends herbivore–plant interaction theory to freshwater ecosystems and offers guidance for aquatic plant restoration and biological management.

## 1. Introduction

Herbivores are key biotic factors shaping plant community structure in both terrestrial and aquatic ecosystems [1–5]. In terrestrial systems, herbivores regulate competitive dynamics through selective feeding, either promoting coexistence by suppressing dominant species or exacerbating exclusion by targeting inferior competitors [6–11]. However, aquatic ecosystems differ fundamentally in environmental conditions, including exponential light attenuation, carbon limitation and buoyancy support, which collectively shape distinct resource use strategies and anti-herbivore defences [12–18].

Unlike terrestrial plants, which commonly rely on lignification and secondary metabolites [19–22], aquatic plants do not. Most aquatic species invest little in structural or chemical defences owing to resource constraints, though exceptions exist (e.g. tannins in watermilfoil and crystal idioblasts in waterlilies) [23,24]. Instead, many aquatic plants reduce consumption by herbivores through morphological adaptations (e.g. greater leaf area and stem elongation) [17,25] or compensate for tissue loss via asexual reproduction, such as ramet production (e.g. *Vallisneria* spp.) or regeneration from stem fragments (e.g. *Elodea* spp. and *Hydrilla verticillata*) [24,26–28]. Crucially, these defence strategies differ markedly among aquatic life forms.

These life-form differences are structured by water depth. Aquatic plants comprise emergent plants with leaves that photosynthesize in air, floating-leaved plants rooted in sediment but with leaves at the water–air interface, and completely submerged plants with leaves photosynthesizing underwater [16]. These life forms occupy distinct vertical positions in the water column and vary in morphology, environmental tolerance and resource acquisition capacity, generating complex competitive dynamics [29]. Importantly, these defence strategies differ markedly among aquatic life forms: emergent plants are largely inaccessible to aquatic herbivores because of their aerial foliage, whereas floating-leaved species typically have tougher tissues than submerged species, making them less palatable.

In shallow littoral zones, floating-leaved species overlap with submerged species [30]. Floating-leaved plants intercept photosynthetically active radiation through dense surface canopies, imposing strong light limitation on underlying submerged plants [30,31]. Conversely, submerged species maintain competitive advantages at deeper water through enhanced light-use efficiency under low light and rapid clonal expansion [32,33]. However, this competitive balance is increasingly disrupted across global lake ecosystems, where submerged plants are declining, and floating-leaved plants are expanding [34–36]. Although abiotic drivers, including eutrophication and hydrological alteration, are widely implicated in this transition [35,37,38], the role of biotic drivers (e.g. herbivorous fish) in mediating these competitive outcomes remains insufficiently explored, especially given the pronounced differences in anti-herbivore defences between these two life forms.

Here, we evaluated how herbivorous fish influence competition between floating-leaved and submerged plants using a mesocosm experiment with four aquatic plant species and one fish species. We estimated intra- and interspecific competition coefficients (*α*) and fish effects (*b*, *m*) within a Ricker competition framework. We hypothesize that (H1) floating-leaved plants possess competitive advantages over submerged species owing to structural and trait differences, and (H2) selective fish grazing on the less-defended submerged plants further weakens their competitiveness, amplifying floating-leaved plant dominance.

## 2. Material and methods

### (a) Experimental material

Plant seedlings used in the experiment were obtained from Wuhan Botanical Garden (30°33′ N, 114°24′ E, altitude 19–35 m). Floating-leaved and submerged plants typically inhabit littoral zones, where they exhibit niche overlap and resource competition [30,39]. Given resource constraints, we selected four species that capture key functional variation within and between life forms: two floating-leaved species, *Nymphoides peltata* (FA) and *Nymphoides indica* (FB), and two submerged species, *Vallisneriadenseserrulata* (SA) and *Myriophyllum spicatum* (SB). While more species would be preferable, this trade-off between species richness and replication is common in experimental ecology; our design reasonably balances species number with life form and functional-type representation (supplementary material, figure S1 and table S1).

Floating-leaved plants represent a group with strongly convergent leaf functional traits [40,41]. We selected two congeners, *N. peltata* and *N. indica*, as representatives of this life form. Both species can reproduce sexually and vegetatively; they differ in floral colour, with *N. peltata* bearing yellow flowers and *N. indica* bearing white flowers [31,42]. Functionally, both exhibited higher relative maximum electron transport rates (rETR_max_: 120–260 µmol m⁻^2^ s⁻^1^) than submerged plants, reflecting strong photosynthetic capability (supplementary material, table S1). The two species also differ in specific leaf area (SLA) and the ratio of carbon to nitrogen (C/N): *N. peltata* has SLA of 240–440 cm^2^ g⁻^1^ and C/N of 18.2 ± 3.7, whereas *N. indica* has SLA of 270–490 cm^2^ g⁻^1^ and C/N of 25.5 ± 6.5 (supplementary material, table S1).

The two submerged species are widely distributed in the middle and lower reaches of the Yangtze River. They exhibited moderate relative maximum electron transport rates (rETR_max_: 30–90 µmol m⁻^2^ s⁻^1^). *V. denseserrulata* (Hydrocharitaceae) is a dioecious species lacking an erect stem, with broad, basally linear leaves adapted to low-light underwater environments [43]. It reproduces rapidly via clonal ramet production [44,45]. *M. spicatum* (Haloragaceae) is a perennial species characterized by upright shoots and filiform leaves [46]. During the growing season, stems elongate vertically to the water surface and then spread horizontally, creating a dense mat [47].

We selected the herbivorous grass carp (*Ctenopharyngodon idella*), a species that occurs in substantial populations in the Yangtze river [48,49]. Grass carp is the most widely cultured freshwater fish species in the People's Republic of China, owing to its low feed costs, rapid growth and ease of cultivation. Its widespread stocking has also made it a major biological constraint on submerged plant restoration in Chinese lakes [50,51]. Juvenile fish used in this experiment were purchased from the Guangdong Aquatic Hatching and Aquaculture Plant.

### (b) Experimental design

From April to August 2025, we conducted a mesocosm experiment in a greenhouse of the Wuhan Botanical Garden. The experimental system consisted of plastic buckets (upper diameter × height: 0.65 × 0.82 m^2^) filled with tap water (total nitrogen concentration: 1.62 ± 0.23 mg l^–1^, total phosphorus concentration: 0.06 ± 0.01 mg l^–1^). The substrate was pastoral soil (TC: 0.81 ± 0.009%, TN: 0.015 ± 0.003% and TP: 0.43 ± 0.03 mg g^–1^). Tap water was used to standardize water chemistry across replicates and to minimize interference from phytoplankton. The combined nutrient pool from the water column and sediment was sufficient to support vigorous growth of all four macrophyte species throughout the experiment, with no visible symptoms of nutrient limitation [52,53].

Seedlings of each plant species were individually cultivated in plastic cups (upper diameter × height: 0.12 × 0.11 m^2^), with one seedling per cup. The cups were filled with soil to a depth of 10 cm (figure 1A). Following a one-month preculture period, plants exhibiting vigorous growth and comparable sizes were selected for subsequent experiments. The average plant length of the selected individuals was 44.7 ± 5.8 cm, and the average plant weight was 7.3 ± 2.4 g.

**Figure 1.**
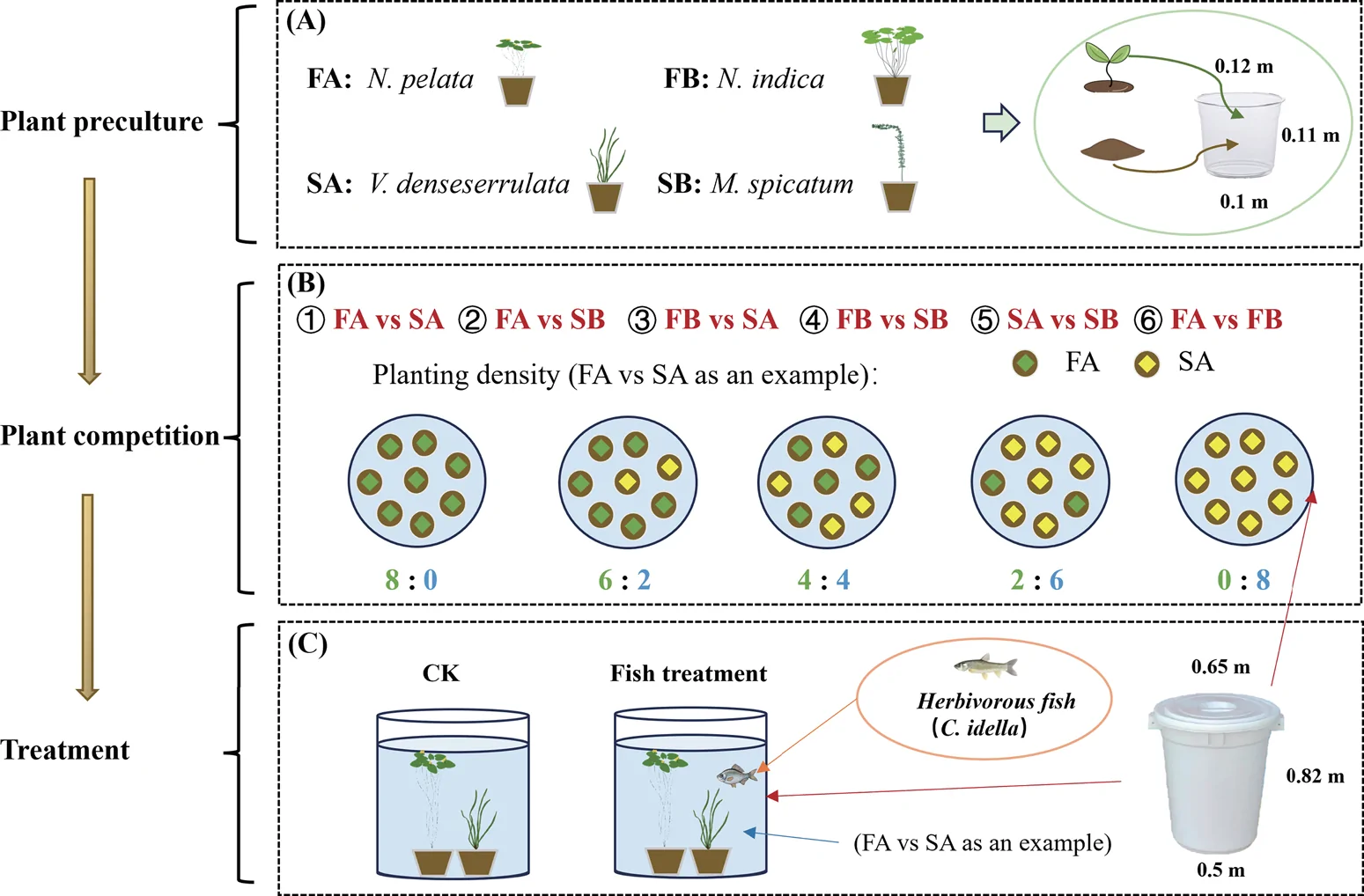
Schematic of the competition experiment design, including (A) plant cultivation method, (B) competition experiment design, and (C) experimental treatments and experimental system. FA: *N. peltata*, FB: *N. indica*, SA: *V. denseserrulata* and SB: *M. spicatum*. CK: the control without fish.

Subsequently, the selected plants were transferred into plastic buckets, with each bucket containing eight plants. To investigate the competition between floating-leaved and submerged plants, we established the following competitive groups: FA versus SA, FA versus SB, FB versus SA and FB versus SB. To explore competition between the two species within a single life form, we set up the following treatments: FA versus FB and SA versus SB. To quantify interspecific competition among plants, six competitive groups were designed to have identical competitive replacement sequences [54,55]. Taking FA versus SA as an example, the plants within the bucket were planted as follows: 8 FA, 6 FA + 2 SA, 4 FA + 4 SA, 2 FA + 6 SA, and 8 SA (figure 1B). Within each bucket, plants were randomly arranged.

Finally, two treatments (a fish-free control and a fish treatment) were established for all competitive experiment designs to assess the impact of herbivorous fish on aquatic plant competition (figure 1C). For the fish treatment, one individual of grass carp (body length: 11–13 cm) was introduced into each mesocosm. Fish stocking density followed Zhen *et al.* [50]. Thus, this experiment comprised six plant competitive groups × five competition sequences × two experimental treatments × five replicates. Single-species mesocosms were shared in different plant competition designs (e.g. 8FA, 8FB, 8SA, and 8SB), and thus a total of 220 mesocosms were constructed. Following a one-month preculture, fish were added on 7 June 2025, and the experiment ended on 7 August 2025. We deployed a HOBO data logger (Onset Computer Corporation, MA, USA) at the water surface to record daily irradiance. Maximum values at noon reached 17946 lux, a level suitable for macrophyte growth [56] (supplementary material, figure S2).

Fish survival was monitored every two days throughout the experiment; any dead fish was immediately replaced with an individual of similar size to maintain consistent grazing pressure across replicates. All animal procedures were conducted in accordance with the institutional guidelines of the Wuhan Botanical Garden, Chinese Academy of Sciences (approval no. WBGCAS-2025-001). This study involved non-invasive observation of fish in experimental mesocosms without surgical intervention, anaesthesia or tissue sampling.

### (c) Sample collection

By the end of the experiment, all surviving fish were returned to aquaculture ponds, and all plants were harvested, cleaned and dried at 80°C. Total biomass was measured for each pot and used to calculate interspecific competition coefficients.

Functional traits (SLA, rETRmax, C/N, average leaf area and asexual propagule number) were obtained from direct measurements and published literature to characterize the four species. Detailed analytical methods are provided in the supplementary material. All data and analysis code supporting this study are available on Zenodo [57]. A detailed description of the dataset is provided in the README file within the repository.

### (d) Data analyses

All statistical analyses were performed in R 4.3.1 [58]. To obtain the interaction coefficients for each competitive group of species, we fitted the relative biomass growth (the ratio of final to initial biomass) of the focal individual to the densities of conspecific and heterospecific neighbours using a modified Ricker model [36,59]. The model takes the form as follows:

(2.1) $$\log\left(\frac{B_{i,t}}{B_{i,0}}\right)=\;\lambda_{i}-\alpha_{ii}N_{i,t}-\alpha_{ij}N_{j,t}.$$

In equation (2.1), *B_i_*_,_*_0_* and *B_i_*_,_*_t_* are the biomass of an individual of species *i* at the beginning and end of the experiment, respectively. *λ_i_* is the potential biomass growth of species *i* in the absence of neighbours; here, *α_ii_* represents the coefficient of intraspecific competition of species *i*; *α_ij_* is the interaction coefficient describing the *per capita* effects of species *j* on species *i*. *N_i_*_,_*_t_*/*N_j_*_,_*_t_* denotes the density of species *i*/*j* at time *t*.

Because *N_i_*_,_*_t_* + *N_j_*_,_*_t_* = 8, we can obtain the following model:

(2.2) $$\log\left(\frac{B_{i,t}}{B_{i,0}}\right)=\;\lambda_{i}-8\alpha_{ij}-\left(\alpha_{ii}-\alpha_{ij}\right)N_{i,t}.$$

Following equation (2.2), the competitive coefficient difference (*α_ii_* – *α_ij_*) was estimated directly via nonlinear least squares as a single composite parameter, without requiring separate identification of the individual coefficients (*α_ij_*). If *α_ii_* – *α_ij_* > 0, intraspecific competition of species *i* is greater than interspecific competition from species *j*, indicating that species *i* experiences stronger intraspecific competition and is more competitive than species *j*. Conversely, if *α_ii_* – *α_ij_* < 0, interspecific competition from species *j* exceeds intraspecific competition of species *i*, indicating that species *j* has a greater competitive advantage.

Subsequently, to quantify the effects of herbivorous fish on plant competition, we extended the baseline Ricker model by incorporating a fish treatment term:

(2.3) $$\frac{B_{i,t}}{B_{i,0}}\;=\;\lambda_{i}\exp[\left(a+bT\right)N_{j,t}]-mT,$$

where *T* is the fish treatment (0 = no fish, 1 = with fish), *a* is the baseline interspecific competition coefficient, *b* represents the modification of competition by fish and *m* is the direct effect coefficient of grass carp. When *T* = 0, the model reduces to the baseline competition model; when *T* = 1, the competition coefficient becomes (*a* + *b*), with an additional direct grazing loss *m*.

In pairwise combinations (species *i* versus species *j*), species *i* was the focal species. A positive *b* indicates that fish intensify competition exerted by species *j* on species *i*, whereas a negative *b* indicates weakened competition; *m* > 0 means that fish inhibit the growth of species *i* and *m* < 0 means that fish benefit species *i*.

## 3. Results

### (a) Competitive relationships among aquatic plants

Floating-leaved plants versus submerged plants: floating-leaved species showed stronger intraspecific than interspecific competition. When FA and FB served as species *i*, and SA and SB as species *j*, respectively, we consistently found that *α_ii_ – α_ij_ >* 0 (range: 0.02–0.133). Conversely, submerged species were more affected by interspecific competition (*α_jj_* – *α_ji_* < 0, range: –0.038 to –0.133) (figure 2A).

**Figure 2.**
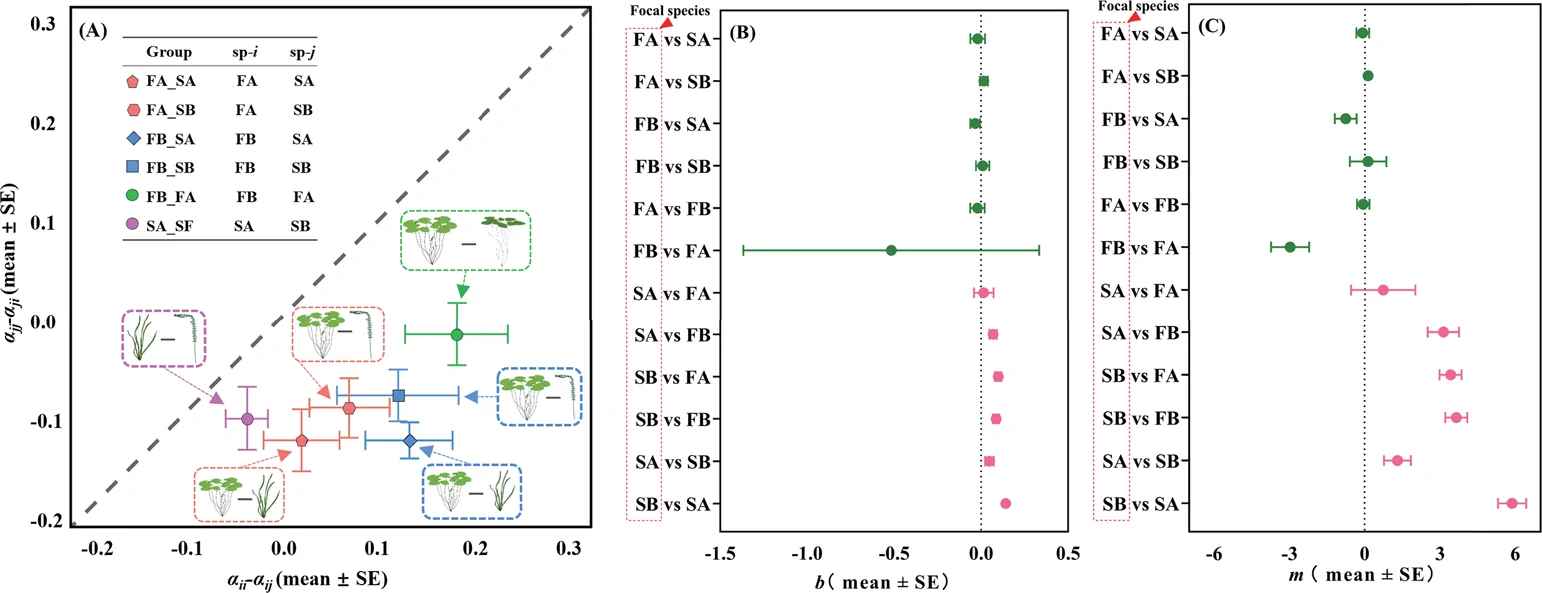
(A) The difference between intraspecific (*α_ii_*, *α_jj_*) and interspecific (*α_ij_*, *α_ji_*) competition coefficients for each competition combination. (B) Effects (*b*) of grass carp on the competition with plants. (C) Direct effects (*m*) of grass carp on aquatic plants. Where *α_ii_* or *α_jj_* represents the coefficient of intraspecific competition of species *i* and species *j*, the coefficient *α_ij_* represents the interspecific competition of species *j* against species *i*, and *α_ji_* represents the interspecific competition of species *i* against species *j*. *b* is the competition modification coefficient, indicating how fish alter the competitive effect of the neighbour species on the focal species. *m* represents the herbivorous fish's direct effect on the focal species. In FA versus SA, FA served as the focal species. FA, *N. peltata*; FB, *N. indica*; *SA*, *V. denseserrulata*; SB, *M. spicatum*.

For the two floating-leaved plants, FB (as species *i*) versus FA (as species *j*), *α_ii_* – *α_ij_* = 0.183 ± 0.054, while *α_jj_* – *α_ji_* = –0.021 ± 0.033. Between the two submerged species, SA (as species *i*) versus SB (as species *j*), *α_ii_* – *α_ij_* = –0.038 ± 0.022 and *α_jj_* – *α_ji_* = –0.110 ± 0.033.

### (b) Effects of herbivorous fish on aquatic plant competition

During the experiment, we observed clear evidence of fish grazing on the leaves of all four aquatic plants (supplementary material, figure S1). Submerged species were heavily grazed, with most leaves and young shoots of SA and SB consumed by grass carp.

Fish grazing effects differed between submerged and floating-leaved plants (figure 2B,C). When floating-leaved species served as the focal species, both the competition modification coefficient (*b*) and the direct effect coefficient (*m*) were generally non-significant. The exception was FB competing against SA or FA (FB as the focal species): here, *m* was significantly negative for FB. Conversely, when submerged species served as the focal species, *b* and *m* were predominantly positive in most comparisons. This pattern held for both SA and SB, except when SA versus FA, where the fish effect was only marginal.

## 4. Discussion

Our experimental evidence amplified that floating-leaved plants showed a competitive advantage over submerged species, and that herbivorous fish amplify this asymmetry. These findings suggest that biotic factors could be important in aquatic plant community succession.

### (a) Asymmetric competition between floating-leaved and submerged plants

Floating-leaved species maintained consistent competitive advantages even under high-transparency conditions, likely owing to their superior light capture and rapid canopy formation. Their high specific leaf area and photosynthetic capacity (supplementary material, table S1) enabled 100% surface coverage within two months, intercepting photosynthetically active radiation and shading underlying submerged vegetation [60,61]. Consequently, submerged species exhibited a decline in biomass throughout the experiment. This pattern held even within the same life form: *N. indica* outcompeted *N. peltata* through greater specific leaf area, illustrating that small functional trait differences can drive competitive outcomes [42,62]. Our results suggest that, among the species tested, such asymmetric competition may ultimately lead to the decline of submerged plants and their local displacement by floating-leaved species.

### (b) Herbivory amplifies the competitive dominance of floating-leaved plants

Submerged and floating-leaved species differed markedly in susceptibility to grass carp grazing. Floating-leaved species possessed structural defences: cuticular wax layers and calcium oxalate crystals in leaf idioblasts rendered their tissues physically resistant to herbivory [63–65]. Grass carp also altered leaf traits in floating-leaved species, with increased average leaf area under herbivory (supplementary material, figure S3A), reflecting the morphological adaptive strategies noted above [17,25]. By contrast, submerged plants lacked comparable defences; their soft, hydrated tissues were readily consumed and their stems were repeatedly severed (supplementary material, figure S1), drastically reducing photosynthetic area and suppressing growth.

This asymmetric herbivory reshaped competitive balance via two quantifiable pathways. Floating-leaved and submerged plants occupy different vertical positions in the water column, leading to asymmetric light competition [30]. Grass carp grazing intensified this competitive asymmetry: direct negative effects on submerged plants (*m* > 0) contrasted with neutral or positive effects on floating-leaved species (*m* ≤ 0) (figure 2C). Additionally, positive *b* values indicated that herbivory intensified the competitive effect of floating-leaved plants on submerged species (figure 2B). This differential impact reduced competitive resistance from submerged macrophytes, allowing floating-leaved canopies to expand. Fish herbivory therefore operated through two pathways: direct suppression of submerged plant growth, and indirect modification of the interspecific competitive balance. The overall outcome was a substantial shift in competitive balance towards floating-leaved dominance.

These results demonstrated that herbivory reshapes aquatic plant communities through higher-order interactions rather than simple biomass removal. Such interactions occur when a third species (e.g. grass carp) alters the interaction between two other species (e.g. submerged and floating-leaved plants) [66,67]. Classical herbivory theory predicts that selective feeding on inferior competitors reinforces the dominance of superior species, a pattern well documented in terrestrial systems [7–9]. Our findings extend this framework to aquatic plants: preferential removal of submerged biomass by grass carp weakens the competitive capacity of submerged species. By quantifying both the competition modification effect (*b*) and the direct fish effect (*m*) within the Ricker model, our study provides a quantitative approach for detecting such higher-order herbivore–plant interactions in aquatic ecosystems.

### (c) Ecological implications

While limited in scale, our mesocosm experiment served as a bridge between laboratory experiments and *in situ* lake conditions, identifying competition and herbivory processes relevant to field ecosystems [68]. While abiotic drivers such as eutrophication are well documented [35,37,38], our results highlight selective herbivory as an important complementary biotic driver of community composition. Notably, grazing induced asexual reproduction in submerged species (ramets, stem fragments and lateral branches; supplementary material, figure S3B), consistent with previous work on *M. spicatum* [69]. Although this grazing-induced propagation may enhance submerged plant regeneration and colonization, its long-term consequences for community stability require further study.

These results are particularly relevant to the shallow lakes in the middle and lower Yangtze River basin, where the 2021 fishing ban is restoring fish biomass [70,71]. The competitive shift we observed is not specific to grass carp; rather, it is driven by the interaction between plant defence asymmetry and selective feeding. Any fish that preferentially targets soft, submerged tissues will produce qualitatively similar effects, provided herbivore density reaches a functionally significant level. Future studies should expand the pool of aquatic plant species and test varying fish densities to determine whether these competitive outcomes hold across broader taxonomic and functional ranges.

## Supplementary Material



## Data Availability

All data and analysis code supporting this study are available on Zenodo [57]. The dataset includes biomass of four macrophyte species across competition and herbivory treatments, leaf functional traits (C/N ratio, average leaf area), asexual propagule numbers, surface light intensity records and R code for nonlinear least-squares fitting of the Ricker competition model. A detailed description of the dataset is provided in the README file within the repository. Supplementary material is available online [72].

## Declaration of AI use

During the preparation of the manuscript, the authors used KIMI AI (developed by Moonshot AI, version K2.6) for language refinement, grammar checking and reference formatting. The AI tool was used from 1 February 2026 to 22 May 2026. Specifically, the authors employed AI tools for the following tasks: (i) language refinement: using AI to polish selected sentences to enhance the fluency of English writing; (ii) format verification: using AI to check grammatical and spelling errors; (iii) reference organization: using AI to assist in managing reference formats.
