# Supplementary material for: Do herbivorous fish amplify the competitive dominance of floating-leaved plants over submerged plants?
Source: Biol Lett. 2026 Oct 28;22(10):20260343. doi: 10.1098/rsbl.2026.0343 (PMC13725520; doi:10.1098/rsbl.2026.0343)
Supplement: Supplementary file 1 [file rsbl.2026.0343.zip › rsbl.2026.0343/rsbl20260343_si_001.docx]

**Title**: Do herbivorous fish amplify the competitive dominance of floating-leaved plants over submerged plants?

**Authors:** Manli Xia, ZhongMing Ye, Guoqing Quan, Wei Liu, Hui Ma, Wei Li, Yu Cao.

**Corresponding author:** Yu Cao

**Declaration of AI use**

During the preparation of this manuscript, the authors used KIMI AI (developed by Moonshot AI, version K2.6) for language refinement, grammar checking, and reference formatting. The AI tool was used from 1 February 2026 to 22 May 2026. Specifically, the authors employed AI tools for the following tasks:

1) Language refinement: Using AI to polish selected sentences to enhance the fluency of English writing.

2) Format verification: Using AI to check grammatical and spelling errors.

3) Reference organization: Using AI to assist in managing reference formats.
