# Supplementary material for: Do herbivorous fish amplify the competitive dominance of floating-leaved plants over submerged plants?
Source: Biol Lett. 2026 Oct 28;22(10):20260343. doi: 10.1098/rsbl.2026.0343 (PMC13725520; doi:10.1098/rsbl.2026.0343)
Supplement: Supplementary file 1 [file rsbl.2026.0343.zip › rsbl.2026.0343/rsbl20260343_si_002.docx]

**Supplementary Material**

**Title**: Do herbivorous fish amplify the competitive dominance of floating-leaved plants over submerged plants?

**Authors:** Manli Xia, ZhongMing Ye, Guoqing Quan, Wei Liu, Hui Ma, Wei Li, Yu Cao.

**Corresponding author:** Yu Cao

**Table**

**Table S1:** Physiological trait characteristics of common floating-leaved and submerged plants in lakes of the middle-lower Yangtze River basin

| Characteristics | Life form | rETRmax (μmol m^-2^ s^-1^) | SLA (cm^2^ g^-1^) | C/N |
| --- | --- | --- | --- | --- |
| *Nymphoides hydrophylla* | Floating-leaved | 100-220^[1]^ | 500-900^[1]^ | / |
| ***N. peltata*** | Floating-leaved | 170 ~ 260^[1]^ | 240 ~ 404^[1]^ | 18.2 ± 3.7 |
| ***N. indica*** | Floating-leaved | 120 ~230^[1]^ | 270 ~ 490^[1]^ | 25.5 ± 6.5 |
| *Trapa bispinosa* | Floating-leaved | / | 200-500^[2]^ | / |
| *Vallisneria natans* | Submerged | 15 ~ 30^[3]^ | 740 ~ 860^[4]^ | / |
| ***V. denseserrulata*** | Submerged | 31 ~ 48^[9]^ | 600 ~ 1000^[10]^ | 16.1 ± 2.0 |
| *Hydrilla verticillata* | Submerged | 24 ~ 34 ^[3, 5]^ | 2700-5200^[2, 4]^ | / |
| ***M. spicatum*** | Submerged | 30 ~ 90^[6, 7]^ | 1170 ~ 1560^[4]^ | 19.9 ± 5.8 |
| *Ceratophyllum demersum* | Submerged | 37 ~ 68^[5, 8]^ | 1140 ~ 1550^[4]^ | / |
| *Potamogeton wrightii* | Submerged | 32 ~ 41^[5]^ | 1070 ~ 1170^[4]^ | / |
| *P. maackianus* | Submerged | / | 1340 ~ 1500^[4]^ | / |
| *P. pectinatus* | Submerged | / | 810 ~ 930^[4]^ | / |

*Values for the maximum relative electron transport rate (rETRmax) and specific leaf area (SLA) were obtained from published literature. The ratio of carbon and nitrogen (C/N) was determined from measurements. At the end of the experiment, all plants were collected, washed, oven-dried at 80 °C to constant mass, ground, and analyzed for total carbon and nitrogen content using an elemental analyzer.

**References in Table S1**

[1] Liu W, Xia M, Mangoi C et al. 2026 Distinct adaptive strategies of three floating-leaved species in Nymphoides across water depth variations: evidence from morphology, hydraulics and photosynthesis. *Wetlands* 46(4), 37.

[2] Liu H, Liu G, Xing W. 2021 Functional traits of submerged macrophytes in eutrophic shallow lakes affect their ecological functions. *Sci. Total. Environ.* 760, 143332.

[3] Gao X, Liu H, Liu G, Huang W, Xing W. 2024 How functional traits of submerged macrophytes response to underwater light quality?. Hydrobiologia 851(21), 5043-5058.

[4] Fu H, Yuan G, Cao T, Ni L, Li W, Zhu G. 2012 Relationships between relative growth rate and its components across 11 submersed macrophytes. *J. Freshwater Ecol.* 27(4), 471-480.

[5] Jiang H, Zhang Y, Yin L et al. 2018 Diurnal changes in photosynthesis by six submerged macrophytes measured using fluorescence. *Aquat. Bot.* 149, 33-39.

[6] Liu H, Liu G, Xing W. 2021 Functional traits of submerged macrophytes in eutrophic shallow lakes affect their ecological functions. *Sci. Total Environ.* 760, 143332.

[7] Xu H, Li W, Fu W et al. 2025 Aquatic plant Myriophyllum spicatum displays contrasting morphological, photosynthetic, and transcriptomic responses between its aquatic and terrestrial morphotypes. *Photosynth. Res.* 163(1), 15.

[8] Chotikarn P, Pramneechote P, Sinutok S 2022. Photosynthetic responses of freshwater macrophytes to the daily light cycle in Songkhla Lagoon. *Plants* 11(21), 2806.

[9] Gao F, Zhang YM, Yang F et al. 2017 Growth and photosynthetic fluorescence characteristics responses of four submersed macrophytes to rising water level. *J. Ecol. Rural Environ.* 33(4), 341–348.

[10] Zhi Y, Wang W, Li W et al. 2024 Increased nutrient levels induce different allocation strategies between canopy-forming and rosette-like submerged macrophytes. *Water* 16(22), 3196.

**Figure**

**Figure S1:** Morphological characteristics of *Nymphoides peltata*, *N. indica*, *Myriophyllum spicatum*, and *Vallisneria denseserrulata*, and performance of leaf feeding by fish. In the leaf characteristics section, we show two leaf states for each species: intact leaf morphology and leaf morphology after grazing by herbivorous fish (indicated by red arrows). The results of the four species after feeding were as follows: *N. peltata* and *N. indica*, leaf edges were grazed; *M. spicatum*, most segments of leaves were grazed, and stems were complete; *V. denseserrulata*, shortened stripe leaves after grazing.

**Figure S2: Dynamics of water surface light intensity during the experiment (15 June–7 August 2025)**

**Figure S3:** Average leaf area of FA and FB (A), asexual propagules number of four aquatic species (B) under different treatments. FA: *Nymphoides peltata*, FB: *N. indica*, SA: *Vallisneria denseserrulata*, and SB: *Myriophyllum spicatum* (SB).

*At the end of the experiment, leaf area was counted for FA and FB, ramets were counted for FA and SA, ramets and fragments were counted for FB, and stem fragments and branches were counted for SB, in single-species mesocosms (e.g., four mesocosms of FA, FB, SA, and SB, each for both CK and Fish treatment).
