# Supplementary material for: Do herbivorous fish amplify the competitive dominance of floating-leaved plants over submerged plants?
Source: Biol Lett. 2026 Oct 28;22(10):20260343. doi: 10.1098/rsbl.2026.0343 (PMC13725520; doi:10.1098/rsbl.2026.0343)
Supplement: Supplementary file 1 [file rsbl.2026.0343.zip › rsbl.2026.0343/rsbl20260343_si_003.docx]

**Title**: Do herbivorous fish amplify the competitive dominance of floating-leaved plants over submerged plants?

**Authors:** Manli Xia, ZhongMing Ye, Guoqing Quan, Wei Liu, Hui Ma, Wei Li, Yu Cao.

**Corresponding author:** Yu Cao

**Authors’ contributions**

MLX conceived of the study, participated in the conceptualization and methodology, carried out the investigation and data curation, performed formal analysis and visualization, and drafted the manuscript; ZMY participated in the conceptualization and methodology, and drafted the manuscript; GQQ, WL and HM carried out the investigation and data curation, and edited the manuscript; WLi conceived of the study, participated in the conceptualization, administered the project and supervised the study, and helped draft the manuscript; YC conceived of the study, participated in the conceptualization and formal analysis, performed visualization, administered the project and supervised the study, and drafted the manuscript. All authors gave final approval for publication and agree to be held accountable for the work performed therein.
