# Supplementary material for: Do herbivorous fish amplify the competitive dominance of floating-leaved plants over submerged plants?
Source: Biol Lett. 2026 Oct 28;22(10):20260343. doi: 10.1098/rsbl.2026.0343 (PMC13725520; doi:10.1098/rsbl.2026.0343)
Supplement: Supplementary file 1 [file rsbl.2026.0343.zip › rsbl.2026.0343/rsbl20260343_si_004.docx]

**Title**: Do herbivorous fish amplify the competitive dominance of floating-leaved plants over submerged plants?

**Authors:** Manli Xia, ZhongMing Ye, Guoqing Quan, Wei Liu, Hui Ma, Wei Li, Yu Cao.

**Corresponding author:** Yu Cao

**Ethics statement**
