# Supplementary material for: Do herbivorous fish amplify the competitive dominance of floating-leaved plants over submerged plants?
Source: Biol Lett. 2026 Oct 28;22(10):20260343. doi: 10.1098/rsbl.2026.0343 (PMC13725520; doi:10.1098/rsbl.2026.0343)
Supplement: Supplementary file 1 [file rsbl.2026.0343.zip › rsbl.2026.0343/rsbl20260343_si_005.docx]

**Article type:** Research Articles

**Title**: Do herbivorous fish amplify the competitive dominance of floating-leaved plants over submerged plants?

Manli Xia^1^, ZhongMing Ye^1^, Guoqing Quan^1, 2^, Wei Liu^1, 2^, Hui Ma^1, 3^, Wei Li ^4, 5, 6^, Yu Cao^1 *^

^1^ Research Center of Aquatic Plant and Watershed Ecology, Wuhan Botanical Garden, Chinese Academy of Sciences, Wuhan 430074, China

^2^ College of Horticulture and Gardening, Yangtze University, Jingzhou, China

^3^ College of Resources and Environment, Huazhong Agricultural University, Wuhan, China

^4^ Yani Wetland Ecosystem Positioning Observation and Research Station, Xizang University, Lhasa 850000, China

^5^ Key Laboratory of Biodiversity Conservation and Ecological Restoration, Shannan 856000, China

^6^ Nyingchi National Botanical Garden, Nyingchi 860000, China

* Yu Cao.

Email: [caoyu@wbgcas.cn](mailto:caoyu@wbgcas.cn)

**Abstract word count:** 151 words

**Main text word count:** 3383 words

**Number of references:** 74

**Number of figures:** 2

**Abstract:**

Growing interest in the relationship between herbivorous fish and aquatic plants has revealed multiple pathways by which fish can influence aquatic plant biomass and coverage. However, the impact of herbivorous fish on species competition and community assembly in aquatic plants remains poorly understood. Here, based on a mesocosm experiment with four aquatic plant species (two floating-leaved and two submerged) and one herbivorous fish, we quantified competition coefficients among plant species and fish effects with a modified Ricker model. Floating-leaved plants exhibited stronger intraspecific than interspecific competition, indicating competitive superiority over submerged species. Crucially, selective grazing by herbivorous fish on submerged plants indirectly facilitated the dominance of floating-leaved species, exacerbating competitive asymmetry. These results demonstrate that herbivory indirectly affects aquatic plant community structure, not simply by removing biomass but also via higher-order interactions. Our research extends herbivore-plant interaction theory to freshwater ecosystems and offers guidance for aquatic plant restoration and biological management.

**Keywords:** Asymmetric competition, Biotic interactions, Community succession, Selective herbivory

**Main Text**

1. **Introduction**

Herbivores are key biotic factors shaping plant community structure in both terrestrial and aquatic ecosystems (Chesson & Kuang, 2008; de Vries et al., 2017; Agrawal & Maron, 2022; Carroll & MacDougall, 2024; Lucas et al., 2025). In terrestrial systems, herbivores regulate competitive dynamics through selective feeding, either promoting coexistence by suppressing dominant species or exacerbating exclusion by targeting inferior competitors (Carson & Root, 2000; Preisser et al., 2008; Kim et al., 2013; Koerner et al., 2018; Schmidt et al., 2020; Eskelinen et al., 2022). However, aquatic ecosystems differ fundamentally in environmental conditions, including exponential light attenuation, carbon limitation, and buoyancy support, which collectively shape distinct resource use strategies and anti-herbivore defenses (Lacoul & Freedman, 2006; Bornette & Puijalon, 2011; Fisher et al., 2012; Craine & Dybzinski, 2013; Li, 2014; He et al., 2019; Roberts & Hanan, 2025).

Unlike terrestrial plants, which commonly rely on lignification and secondary metabolites (Coley & Barone, 1996; Gatehouse, 2002; Vermeij, 2016; Grandi et al., 2024). Most aquatic species invest little in structural or chemical defenses due to resource constraints, though exceptions exist (e.g., tannins in watermilfoil and crystal idioblasts in waterlilies) (Fornoff & Gross, 2014; Kuntz et al., 2014). Instead, many aquatic plants reduce herbivore consumption through morphological adaptations (e.g., greater leaf area, stem elongation) (Li et al., 2014; Dong et al., 2017) or compensate for tissue loss via asexual reproduction, such as ramet production (e.g., *Vallisneria* spp.) or regeneration from stem fragments (e.g., *Elodea* spp., *Hydrilla verticillata*) (Santamaría, 2002; Kuntz et al., 2014; Coughlan et al., 2022; Thomaz, 2025). Crucially, these defense strategies differ markedly among aquatic life forms.

These life-form differences are structured by water depth. Aquatic plants comprise emergent plants with leaves that photosynthesize in air, floating-leaved plants rooted in sediment but with leaves at the water-air interface, and completely submerged plants with leaves photosynthesizing underwater (Lacoul & Freedman, 2006). These life forms occupy distinct vertical positions in the water column and vary in morphology, environmental tolerance, and resource acquisition capacity, generating complex competitive dynamics (Hu et al., 2021). Crucially, these defense strategies differ markedly among aquatic life forms: emergent plants are largely inaccessible to aquatic herbivores because of their aerial foliage, whereas floating-leaved species typically have tougher tissues than submerged species, making them less palatable.

In shallow littoral zones, floating-leaved species overlap with submerged species (Chao et al., 2025). Floating-leaved plants intercept photosynthetically active radiation through dense surface canopies, imposing strong light limitation on underlying submerged plants (Larson, 2007; Chao et al., 2025). Conversely, submerged species maintain competitive advantages at deeper water through enhanced light-use efficiency under low light and rapid clonal expansion (Li et al., 2020; Chen et al., 2022). However, this competitive balance is increasingly disrupted across global lake ecosystems, where submerged plants are declining, and floating-leaved plants are expanding (Liu et al., 2025; Luo et al., 2025; Wu et al., 2025). Although abiotic drivers, including eutrophication and hydrological alteration, are widely implicated in this transition (Regmi et al., 2021; Jin et al., 2024; Luo et al., 2025), the role of biotic drivers (e.g., herbivorous fish) in mediating these competitive outcomes remains insufficiently explored, especially given the pronounced differences in anti-herbivore defenses between these two life forms.

Here, we evaluated how herbivorous fish influence competition between floating-leaved and submerged plants using a mesocosm experiment with four aquatic plant species and one fish species. By estimating intra- and interspecific competition coefficients (*α*) and fish effect (*b*, *m*) within a Ricker competition framework. We hypothesize that (H1) floating-leaved plants possess competitive advantages over submerged species due to structural and trait differences, and (H2) selective fish grazing on the less-defended submerged plants further weakens their competitiveness, amplifying floating-leaved plant dominance.

2. **Materials and methods**
   2.1 **Experimental material**

Plant seedlings used in the experiments were obtained from Wuhan Botanical Garden (30°33′N, 114°24′E, altitude 19 - 35 m). Floating-leaved and submerged plants typically inhabit littoral zones, where they exhibit niche overlap and resource competition (Björn et al., 2022; Chao et al., 2025). Given resource constraints, we selected four species that capture key functional variation within and between life forms: two floating-leaved species, *Nymphoides peltata* (FA) and *N. indica* (FB), and two submerged species, *V. denseserrulata* (SA) and *M. spicatum* (SB). While more species would be preferable, this trade-off between species richness and replication is common in experimental ecology; our design reasonably balances species number with life-form and functional-type representation (Figure S1; Table S1).

Floating-leaved plants represent a group with strongly convergent leaf functional traits (Liu et al., 2023; Chen et al., 2026). We selected two congeners, *N. peltata* and *N. indica*, as representatives of this life form. Both species can reproduce sexually and vegetatively; they differ in floral color, with *N. peltata* bearing yellow flowers and *N. indica* bearing white flowers (Larson, 2007; Liu et al., 2026). Functionally, both exhibited higher relative maximum electron transport rates (rETR_max_: 120-260 μmol m⁻² s⁻¹) than submerged plants, reflecting strong photosynthetic capability (Table S1). The two species also differ in specific leaf area (SLA) and the ratio of carbon and nitrogen (C/N): *N. peltata* has SLA of 240 - 440 cm² g⁻¹ and C/N of 18.2 ± 3.7, whereas *N. indica* has SLA of 270 - 490 cm² g⁻¹ and C/N of 25.5 ± 6.5 (Table S1).

Both selected submerged species are widely distributed in the middle and lower reaches of the Yangtze River. They exhibited moderate relative maximum electron transport rates (rETR_max_: 30 - 90 μmol m⁻² s⁻¹). *V. denseserrulata* (Hydrocharitaceae) is a dioecious species lacking an erect stem, with broad, basally linear leaves adapted to low-light underwater environments (Zhou et al., 2016). It reproduces rapidly via clonal ramets (Liu et al., 2018; Lin et al., 2026). *Myriophyllum spicatum* (Haloragaceae) is a perennial species characterized by upright shoots and filiform leaves (Liu et al., 2021). During the growing season, stems elongate vertically to the water surface and then spread horizontally, creating a dense mat (Titus et al., 1975).

We selected the herbivorous grass carp (*Ctenopharyngodon idella*), a species that occurs in substantial populations in the Yangtze (Xiong et al., 2023; Huo et al., 2026). Grass carp is the most widely cultured freshwater fish species in China, owing to its low feed costs, rapid growth, and ease of cultivation. Its widespread stocking has also made it a major biological constraint on submerged plant restoration in Chinese lakes (Zhen et al., 2018; Zheng et al., 2026). Juvenile fish used in this experiment were purchased from the Guangdong Aquatic Hatching and Aquaculture Plant.

2.2 **Experimental design**

From April to August 2025, we conducted a mesocosm experiment at a greenhouse of the Wuhan Botanical Garden. The experimental system was constructed using plastic buckets (upper diameter × height: 0.65 m × 0.82 m) and filled with tap water (total nitrogen concentration: 1.62 ± 0.23 mg L^-1^, total phosphorus concentration: 0.06 ± 0.01mg L^-1^). The substrate was pastoral soil (TC: 0.81 ± 0.009%, TN: 0.015 ± 0.003%, TP: 0.43 ± 0.03 mg g^-1^). Tap water was used to standardize water chemistry across replicates and to reduce phytoplankton interference. The combined nutrient pool from the water column and sediment was sufficient to support vigorous growth of all four macrophyte species throughout the experiment, with no visible symptoms of nutrient limitation (Zhi et al., 2018; Han et al., 2023).

Seedlings of each plant species were individually cultivated in plastic cups (upper diameter × height: 0.12 m × 0.11 m), with one seedling per cup. The cups were filled with soil to a depth of 10 cm (Figure 1A). Following a one-month pre-cultivation period, plants exhibiting vigorous growth and comparable sizes were selected for subsequent experiments. The average plant length of the selected individuals was 44.7 ± 5.8 cm, and the average plant weight was 7.3 ± 2.4 g.

**Figure 1:** Schematic diagram of the competition experiment design. FA: *N. peltata*, FB: *N. indica*, SA: *V. denseserrulata*, and SB: *M. spicatum*. CK: The control without fish.

Subsequently, the selected plants were transferred into plastic buckets, with each bucket containing eight plants. To investigate the competition between floating-leaved and submerged plants, we established the following competitive groups: FA vs SA, FA vs SB, FB vs SA, and FB vs SB. To explore competition between the two species within a single life form, we set up the following treatments: FA vs FB and SA vs SB. To quantify interspecific competition among plants, six competitive groups were designed to have identical competitive replacement sequences (Jolliffe et al., 1984; Xia et al., 2025). Taking FA vs SA as an example, the plants within the bucket were planted as follows: 8 FA, 6 FA + 2 SA, 4 FA + 4 SA, 2 FA + 6 SA, and 8 SA (Figure 1B). Within each bucket, plants were randomly arranged.

Finally, two treatments (the Control without fish and the fish treatment) were established for all competitive experiment designs to assess the impact of herbivorous fish on aquatic plant competition (Figure 1C). For the fish treatment, one individual of grass carp (body length: 11-13 cm) was introduced into each mesocosm. Fish densities were determined as described in Zhen et al. (2018). Thus, this experiment comprised 6 plant competitive groups × 5 competition sequences × 2 experimental treatments × 5 replicates. Single-species mesocosms were shared in different plant competition designs (e.g., 8FA, 8FB, 8SA, 8SB), and thus a total of 220 mesocosms were constructed. Following a one-month preculture, fish were added on June 7, 2025, and the experiment ended on August 7, 2025. We deployed a HOBO data logger (Onset Computer Corporation, MA, USA) at the water surface to record daily irradiance. Maximum values at noon reached 17,946 lux, which is suitable for macrophyte growth (Sand-Jensen & Madsen, 1991) (Figure S2).

Fish survival was monitored every two days throughout the experiment; any mortality was immediately replaced with an individual of similar size to maintain consistent grazing pressure across replicates. All animal procedures were conducted in accordance with the institutional guidelines of the Wuhan Botanical Garden, Chinese Academy of Sciences (approval number: WBGCAS-2025-001). This study involved non-invasive observation of fish in experimental mesocosms without surgical intervention, anesthesia, or tissue sampling.

2.3 **Sample collection**

By the end of the experiment, all surviving fish were returned to aquaculture ponds, and all plants were harvested, cleaned, and dried at 80°C. Total biomass was measured for each pot and used to calculate interspecific competition coefficients.

Functional traits (SLA, rETRmax, C/N, average leaf area, and asexual propagule number) were obtained from direct measurements and published literature to characterize the four species. Detailed analytical methods are provided in the Supplementary Materials. All data and analysis code supporting this study are available from Zenodo (<https://doi.org/10.5281/zenodo.21964113>) (Xia et al., 2026). A detailed description of the dataset is provided in the README file within the repository.

2.4 **Data analyses**

All statistical analyses were performed in R 4.3.1 (R Core Team, 2025). To obtain the interaction coefficients for each competitive group of species, we fitted the relative biomass growth (the ratio of final to initial biomass) of the focal individual to the densities of its heterospecific neighbors using a modified Ricker model (Ricker, 1954; Wu et al., 2025). The model takes the form as follows:

$\log\left( \frac{B_{i,t}}{B_{i,0}} \right)=\lambda_{i}-\alpha_{ii}N_{i,t}-\alpha_{ij}N_{j,t}$	(1)

In Expression (1), *B_i,0_* and *B_i,t_* are the biomass of an individual of the species *i* at the beginning and end of the experiment, respectively. *λ_i_* is the potential biomass growth of species *i* in the absence of neighbors; here, *α_ii_* represents the coefficient of intraspecific competition of species *i*; *α_ij_* is the interaction coefficient describing the per capita effects of species *j* on species i. *N_i,t_* / *N_j,t_* denotes the density of species *i* / *j* at time t.

Because *N_i,t_* + *N_j,t_* = 8, we can obtain the following model:

$\log\left( \frac{B_{i,t}}{B_{i,0}} \right)=\lambda_{i}-8\alpha_{ij}+(\alpha_{ii}-\alpha_{ij})N_{i,t}$	(2)

Following Expression (2), the competitive coefficient difference (*α_ii_*-*α_ij_*) was estimated directly via nonlinear least squares (nlsLM) as a single composite parameter, without requiring separate identification of the individual coefficients (*α_ij_*). If *α_ii_*-*α_ij_* > 0, intraspecific competition of species *i* is greater than interspecific competition from species *j*, indicating that species *i* experiences stronger intraspecific competition and is more competitive than species *j*. Conversely, if *α_ii_*-*α_ij_* < 0, interspecific competition from species *j* exceeds intraspecific competition of species *i*, indicating that species *j* has a greater competitive advantage.

Subsequently, to quantify the effects of herbivorous fish on plant competition, we extended the baseline Ricker model by incorporating a fish treatment term:

$\frac{B_{i,t}}{B_{i,0}} = \lambda_{i}exp[ (a+bT)N_{j,t}]-mT$	(3)

Where *T* is the fish treatment (0 = no fish, 1 = with fish), *a* is the baseline interspecific competition coefficient, *b* represents the modification of competition by fish, and *m* is the direct effect coefficient of grass carp. When *T*=0, the model reduces to the baseline competition model; when *T*=1, the competition coefficient becomes (*a*+*b*), with an additional direct grazing loss *m*.

In pairwise combinations (species *i* vs. species j), species i was the focal species. A positive *b* indicates that fish intensify competition exerted by species *j* on species *i*, whereas a negative *b* indicates weakened competition; *m* > 0 means that fish inhibit the growth of species *i*, and *m* < 0 means that fish benefit species *i*.

3 **Results**
  3.1 **Competitive relationships among aquatic plants**

Floating-leaved plants vs submerged plants: Floating-leaved species showed stronger intraspecific competition than interspecific competition with submerged species. When FA and FB served as species *i*, and SA and SB as species *j*, respectively, we consistently found that *αii - αij > 0* (range: 0.02 *-* 0.133). Conversely, submerged species were more affected by interspecific competition (*α_jj_* -*α_ji_* < 0, range -0.038 to - 0.133) (Figure 2A).

For the two floating-leaved plants, FB (as species *i*) vs FA (as species *j*), *α_ii_* - *α_ji_* = 0.183 ± 0.054, while *α_jj_* -*α_ji_* = -0.021 ± 0.033. Between the two submerged species, SA (as species *i*) vs SB (as species *j*), *α_ii_* - *α_ji_* = -0.038 ± 0.022, and *α_jj_* -*α_ji_* = -0.110 ± 0.033.

**Figure 2:** (A) The difference between intraspecific (*α_ii_*, *α_jj_*) and interspecific (*α_ij_*, *α_ji_*) competition coefficients between each competition combination. (B) Direct effects (*m*) of grass carp on aquatic plants. Where *α_ii_* or *α_jj_* represents the coefficient of intraspecific competition of species *i* and species *j*, the coefficient *α_ij_* represents the interspecific competition of species *j* against species *i*, and *α_ji_* represents the interspecific competition of species *i* against species *j*. *b* is the competition modification coefficient, indicating how fish alter the competitive effect of the neighbor species on the focal species. *m* represents the herbivorous fish's direct effect on the focal species. When FA vs SA, FA served as the focal species. FA, *N. peltata*; FB, *N. indica*; *SA*, *V. denseserrulata*; SB, *M. spicatum*.

3.2 **Effects of herbivorous fish on aquatic plant competition**

During the experiment, we observed clear evidence of fish grazing on the leaves of all four aquatic plants (Figure S1). Submerged species were grazed to a substantial extent, with most leaves and young shoots of SA and SB consumed by grass carp.

Results indicated that fish grazing effects differed between submerged and floating-leaved plants (Figure 2B). When floating-leaved species served as the focal species, both the competition modification coefficient (*b*) and the direct effect coefficient (*m*) were generally non-significant. The exception was FB competing against SA or FA (FB as the focal species): here, *m* was significantly negative for FB. Conversely, when submerged species served as the focal species, *b* and *m* were predominantly positive in most comparisons. This pattern held for both SA and SB, except when SA vs FA, where the fish effect was only marginal.

4 **Discussion**

Our experimental evidence demonstrated that floating-leaved plants showed a competitive advantage over submerged species, and that herbivorous fish amplify this asymmetry. These findings suggest that biotic factors could be important in aquatic plant community succession.

4.1 **Asymmetric competition between floating-leaved and submerged plants**

Floating-leaved species maintained consistent competitive advantages even under high-transparency conditions, likely owing to their superior light capture and rapid canopy formation. Their high specific leaf area and photosynthetic capacity (Table S1) enabled 100% surface coverage within two months, intercepting photosynthetically active radiation and shading underlying submerged vegetation (Strayer et al., 2003; Seto et al., 2013). Consequently, submerged species exhibited a decline in biomass throughout the experiment. This pattern held even within the same life form: *N. indica* outcompeted *N. peltata* through greater specific leaf area, illustrating that small functional trait differences can drive competitive outcomes (Wu & Yu, 2004; Liu et al., 2026). Our results suggest that, among the species tested, such asymmetric competition may ultimately lead to the decline of submerged plants and their local displacement by floating-leaved species.

4.2 **Herbivory amplifies the competitive dominance of floating-leaved plants**

Submerged and floating-leaved species differed markedly in susceptibility to grass carp grazing. Floating-leaved species possessed structural defenses: cuticular wax layers and calcium oxalate crystals in leaf idioblasts rendered their tissues physically resistant to herbivory (Ronzhina et al., 2010; Klok & van der Velde, 2019; He et al., 2024). Grass carp also altered leaf traits in floating-leaved species, with increased average leaf area under herbivory (Figure S3A), reflecting the morphological adaptive strategies noted above (Li, 2014; Dong et al., 2017). In contrast, submerged plants lacked comparable defenses; their soft, fully hydrated leaves and stems were highly accessible to fish. In contrast, submerged plants lacked comparable defenses; their soft, hydrated tissues were readily consumed, and their stems were repeatedly severed (Figure S1), drastically reducing photosynthetic area and suppressing growth.

This asymmetric herbivory reshaped competitive balance via two quantifiable pathways. Floating-leaved and submerged plants occupy different vertical positions in the water column, leading to asymmetric light competition (Chao et al., 2025). Grass carp grazing intensified this competitive asymmetry: direct negative effects on submerged plants (*m* > 0) contrasted with neutral or positive effects on floating-leaf species (*m* ≤ 0) (Figure 2C). Additionally, positive *b* values indicated that herbivory intensified the competitive effect of floating-leaved plants on submerged species (Figure 2B). This differential impact reduced competitive resistance from submerged macrophytes, allowing floating-leaved canopies to expand. Fish herbivory therefore operated through two pathways: the direct pathway of submerged plant growth suppression, and indirect modification of interspecific competitive balance. The overall outcome was a substantial shift in competitive balance toward floating-leaved dominance.

These results demonstrated that herbivory reshapes aquatic plant communities through higher-order interactions rather than simple biomass removal. Such interactions occur when a third species (e.g., grass carp) alters the interaction between two other species (e.g., submerged and floating-leaved plants) (Levine et al., 2017; Gibbs et al., 2022). Classical herbivory theory predicts that selective feeding on inferior competitors reinforces the dominance of superior species, a pattern well documented in terrestrial systems (Koerner et al., 2018; Kim et al., 2013; Eskelinen et al., 2022). Our findings extend this framework to aquatic plants: preferential removal of submerged biomass by grass carp weakens the competitive capacity of submerged species. By quantifying both the competition modification effect (*b*) and the direct fish effect (*m*) within the Ricker model, our study provides a quantitative approach for detecting such higher-order herbivore–plant interactions in aquatic ecosystems.

4.3 **Ecological implications**

While limited in scale, our mesocosm experiment served as a bridge between laboratory experiments and *in-situ* lake conditions, identifying competition and herbivory processes relevant to field ecosystems (Schaum et al., 2025). While abiotic drivers such as eutrophication are well-documented (Regmi et al., 2021; Jin et al., 2024; Luo et al., 2025), our results highlight selective herbivory as an equally important biotic driver of community composition. Notably, grazing induced asexual reproduction in submerged species (ramets, stem fragments, and lateral branches; Figure S3B), consistent with previous work on *M. spicatum* (Lemoine et al., 2009). Although this grazing-induced propagation may enhance submerged plant regeneration and colonization, its long-term consequences for community stability require further study.

These results are particularly relevant to the shallow lakes in the middle and lower Yangtze River basin, where the 2021 fishing ban is restoring fish biomass (Feng et al., 2026; Xiong et al., 2026). The competitive shift we observed is not specific to grass carp; rather, it is driven by the interaction between plant defense asymmetry and selective feeding. Any fish that preferentially targets soft, submerged tissues will produce qualitatively similar effects, provided herbivore density reaches a functionally significant level. Future studies should expand the pool of aquatic plant species and test varying fish densities to determine whether these competitive outcomes hold across broader taxonomic and functional ranges.

**Funding:**

This study was supported by the National Natural Science Foundation of China (32370388).

**Acknowledgments:**

We are grateful to Charity Kaluki Mangoi, Daoming Cheng, and Chaoyu Tian for their contributions to aquatic plants cultivation and harvesting, to Yuanzhi Li for assistance with data analysis, and to Junfeng Pan for providing plant materials.

**References**

Agrawal AA, Maron JL. 2022 Long-term impacts of insect herbivores on plant populations and communities. *J. Ecol.* 110, 2800–2811.

Björn LO, Middleton BA, Germ M, Gaberščik A. 2022 Ventilation systems in wetland plant species. *Diversity* 2022, 14(7): 517.

Bornette G, Puijalon S. 2011 Response of aquatic plants to abiotic factors: a review. *Aquat. Sci.* 73(1), 1-14.

Carroll O, MacDougall AS. 2024. Facilitation against herbivory is more important than resource‐based niche differences for plant coexistence in a field experiment. *J. Ecol.* 112(6), 1200–1209.

Carson WP, Root RB. 2000 Herbivory and plant species coexistence: community regulation by an outbreaking phytophagous insect. *Ecol. Monogr.* 70, 73–99.

Chao C, Dong L, Yu W et al. 2025 Competitive outcomes between floating‐leaved and submerged plants under eutrophication depend on growth form. *Freshwater Biol.* 70(5), e70050.

Chen J, Chou Q, Ren W et al. 2022 Growth, morphology and C/N metabolism responses of a model submersed macrophyte, *Vallisneria natans*, to various light regimes. *Ecol. Indic.* 136, 108652.

Chesson P, Kuang JJ. 2008 The interaction between predation and competition. *Nature* 456(7219), 235–238. (doi:10.1038/nature07248)

Coley PD, Barone JA. 1996 Herbivory and plant defenses in tropical forests. *Annu. Rev. Ecol. Syst.* 27, 305–335.

Coughlan NE, Armstrong F, Baker‐Arney C et al. 2022. Retention of viability by fragmented invasive *Crassula helmsii*, *Elodea canadensis* and *Lagarosiphon major*. *River. Res. Appl.* 38(8), 1356-1361.

Craine JM, Dybzinski R. 2013 Mechanisms of plant competition for nutrients, water and light. *Funct. Ecol.* 27, 833–840.

de Vries J, Evers JB, Poelman EH. 2017 Dynamic plant-plant-herbivore interactions govern plant growth-defence integration. *Trends Plant Sci.* 22, 329–337.

Dong B, Fu T, Luo F, Yu F. 2017 Herbivory-induced maternal effects on growth and defense traits in the clonal species *Alternanthera philoxeroides*. *Sci. Total. Environ.* 605, 114-123.

Eskelinen A, Harpole WS, Jessen MT, Virtanen R, Hautier Y. 2022 Light competition drives herbivore and nutrient effects on plant diversity. *Nature* 611, 301–305.

Feng K, Tao K, Wang Y et al. 2026 Evaluating restoration success: Ecosystem-level shifts in lake fish biodiversity consistent with China's fishing ban. *Biol. Conserv.* 315, 111733.

Fisher JB, Badgley G, Blyth E. 2012 Global nutrient limitation in terrestrial vegetation. *Global Biogeochem. Cy*. 26, 3.

Fornoff F, Gross EM. 2014 Induced defense mechanisms in an aquatic angiosperm to insect herbivory. *Oecologia* 175, 173–185.

Gatehouse JA. 2002 Plant resistance towards insect herbivores: a dynamic interaction. *New Phytol*. 156, 145–169.

Chen L, Wang J, Cao Y, Chen H et al. 2026 The convergent genomic dynamism for aquatic adaptation in vascular plants. *Curr. Biol.* 36(1), 80-92.

Gibbs T, Levin SA, Levine JM. 2022. Coexistence in diverse communities with higher-order interactions. *P. Natl. Acad. Sci.* 119(43), e2205063119.

Grandi L, Ye W, Clancy MV, et al. 2024 Plant‐to‐plant defence induction in cotton is mediated by delayed release of volatiles upon herbivory. *New Phytol.* 244(6), 2505-2517.

Han H, Wu H, Zhi Y, et al. 2023 Impacts of bisphenol A on growth and reproductive traits of submerged macrophyte *Vallisneria natans*. *Environ. Sci. Pollut. R.* 30(16), 46383-46393.

He H, Li D, Li X et al. 2024 Research progress on the formation, function, and impact of calcium oxalate crystals in plants. *Crystallogr. Rev.* 30, 31–60.

He L, Zhu T, Wu Y, et al. 2019 Littoral slope, water depth and alternative response strategies to light attenuation shape the distribution of submerged macrophytes in a mesotrophic lake. *Front. Plant. Sci.* 10, 169.

Hu J, Yu H, Li Y, et al. 2021 Variation in resource allocation strategies and environmental driving factors for different life‐forms of aquatic plants in cold temperate zones. *J. Ecol.* 109(8), 3046-3059.

Huo R, Zhu Z, Wang P, Song H, Wei S. 2026 Genetic diversity analysis and population structure of grass carp (*Ctenopharyngodon idellus*) in Pearl river, Yangtze river and Heilongjiang river using microsatellite DNA markers. *Mol. Biol. Rep.* 53(1), 29.

Jin L, Wu Q, Xie S, Chen W, Duan C, Sun C, Lauridsen TL. 2024 Phosphorus stoichiometric homeostasis of submerged macrophytes and associations with interspecific interactions and community stability in Erhai Lake, China. *Water Res.* 256, 121575.

Jolliffe PA, Minjas AN, Runecles VC. 1984 A reinterpretation of yield relationships in replacement series experiments. *J. Appl. Ecol.* 21, 227–243.

Kim TN, Underwood N, Inouye BD. 2013 Insect herbivores change the outcome of plant competition through both inter- and intraspecific processes. *Ecology* 94, 1753–1763.

Klok PF, van der Velde G. 2019 Initial decomposition of floating leaf blades of waterlilies: causes, damage types and impacts. *PeerJ* 7, e7158.

Koerner SE, Smith MD, Burkepile DE et al. 2018 Change in dominance determines herbivore effects on plant biodiversity. *Nat. Ecol. Evol.* 2, 1925–1932.

Kuntz K, Heidbüchel P, Hussner A. 2014 Effects of water nutrients on regeneration capacity of submerged aquatic plant fragments. *Ann. Limnol. Int. J. Limnol.* 50, 155–162.

Lacoul P, Freedman B. 2006 Environmental influences on aquatic plants in freshwater ecosystems. *Environ. Rev.* 14, 89–136.

Larson D. 2007 Reproduction strategies in introduced *Nymphoides peltata* populations revealed by genetic markers. *Aquat. Bot.* 86(4), 402–406.

Lemoine DG, Barrat-Segretain MH, Roy A. 2009 Morphological and chemical changes induced by herbivory in three common aquatic macrophytes. *Int. Rev. Hydrobiol.* 94: 282–289.

Levine JM, Bascompte J, Adler PB, Allesina S. 2017. Beyond pairwise mechanisms of species coexistence in complex communities. *Nature* 546(7656), 56-64.

Li L, Ding M, Jeppesen E. 2020 Variation in growth, reproduction, and resource allocation in an aquatic plant, *Vallisneria spinulosa*: the influence of amplitude and frequency of water level fluctuations. *Aquat. Sci.* 82(4), 81.

Li W. 2014 Environmental opportunities and constraints in the reproduction and dispersal of aquatic plants. *Aquat. Bot*. 118, 62–70.

Lin Z, Fu S, Li J, Guan B, Huang X, He H, Yu J. 2026 From environment to biomass: trait-mediated effects on submerged macrophyte biomass in a subtropical shallow restored lake. *BMC Plant Biol.* 26, 592.

Liu H, Liu G, Xing W. 2021 Functional traits of submerged macrophytes in eutrophic shallow lakes affect their ecological functions. *Sci. Total Environ.* 760, 143332.

Liu J, Hou X, Huang H et al. 2025 Transformation of aquatic vegetation in Chinese lakes from 1988 to 2023. *Remote Sens. Environ.* 330, 114999.

Liu W, Xia M, Mangoi C et al. 2026 Distinct adaptive strategies of three floating-leaved species in *Nymphoides* across water depth variations: evidence from morphology, hydraulics and photosynthesis. *Wetlands* 46(4), 37.

Liu Y, Liu H, Baastrup-Spohr L et al. 2023 Allometric relationships between leaf and petiole traits across 31 floating-leaved plants reveal a different adaptation pattern from terrestrial plants. *Ann. Bot.* 131(3), 545-552.

Liu Z, Hu J, Zhong P et al. 2018 Successful restoration of a tropical shallow eutrophic lake: strong bottom-up but weak top-down effects recorded. *Water Res.* 146, 88–97.

Lucas JM, Budny ML, Lemoine NP. 2025 Consumers stabilize grassland ecosystem functions by destabilizing belowground communities under abiotic stress. *Ecology* 106(11), e70254.

Luo J, Duan H, Xu Y et al. 2025 Global trends and regime state shifts of lacustrine aquatic vegetation. *The Innovation* 6(3), 100734.

Preisser EL, Elkinton JS. 2008 Exploitative competition between invasive herbivores benefits a native host plant. *Ecology* 89, 2671–2677.

R Core Team, 2025. R: a Language and Environment for Statistical Computing. R foundation for statistical computing, Vienna, Austria.

Regmi T, Shah DN, Doody TM, Cuddy SM, Shah RDT. 2021 Hydrological alteration induced changes on macrophyte community composition in sub-tropical floodplain wetlands of Nepal. *Aquat. Bot*. 173, 103413.

Ricker WE. 1954 Stock and recruitment. *J. Fish. Res. Board Can.* 11(5), 559–623.

Roberts T, Hanan NP. 2025 Sapflow database reveals density‐dependent competition among woody plants at global scale. *Ecol. Lett.* 28(6), e70167.

Ronzhina DA, Ivanov LA, P'yankov VI. 2010 Chemical composition of leaves and structure of photosynthetic apparatus in aquatic higher plants. *Russ. J. Plant Physiol.* 57(3), 368–375.

Sand-Jensen K, Madsen TV. 1991 Minimum light requirements of submerged freshwater macrophytes in laboratory growth experiments. *J. Ecol.* 749-764.

Santamaría L. 2002 Why are most aquatic plants widely distributed? Dispersal, clonal growth and small-scale heterogeneity in a stressful environment. *Acta Oecol*. 23, 137–154.

Schaum CE, Comeau S, Mullarney JC. 2025 Mesocosms: Bridging the gap between in situ and laboratory studies. *Limnol. Oceanogr.* 70, S7-S13.

Schmidt R, Deising HB, Hensen I, Schädler M, Auge H. 2020 Natural enemies do not contribute to negative frequency-dependence in native and exotic grassland plants. *Perspect. Plant Ecol. Evol. Syst*. 46, 125565.

Seto M, Takamura N, Iwasa Y. 2013 Individual and combined suppressive effects of submerged and floating-leaved macrophytes on algal blooms. *J. Theor. Biol.* 319, 122–133.

Strayer DL, Lutz C, Malcom HM et al. 2003 Invertebrate communities associated with a native (*Vallisneria americana*) and an alien (*Trapa natans*) macrophyte in a large river. *Freshwater Biol.* 48(11), 1938–1949.

Thomaz SM. 2025 Asexual reproduction of aquatic macrophytes via stem fragments: A review on determinants of plant fragmentation and propagule dispersal. *Hydrobiologia* 1-33.

Titus JE, Goldstein RA, Adams MS et al. 1975 A production model for *Myriophyllum spicatum* L. *Ecology* 56(5), 1129–1138.

Vermeij GJ. 2016 Plant defences on land and in water: why are they so different?. *Ann. Bot-London*. 117(7), 1099-1109.

Wu T, Li Y, Cadotte MW et al. 2025 Plant invasion decreases the likelihood of community persistence through asymmetric competition. *Ecol. Lett.* 28(8), e70154.

Wu Z, Yu D. 2004 The effects of competition on growth and biomass allocation in *Nymphoides peltata* (Gmel.) O. Kuntze growing in microcosm. *Hydrobiologia* 527, 241–250.

Xia M, Liu W, Tian C et al. 2025 Apparent competition between two submerged macrophytes via fish: evidence from a mesocosm study. *Freshwater Biol.* 70(10), e70116.

Xia M, Ye Z, Quan G, Liu W, Ma H, Li W, Yu C. 2026. Do herbivorous fish amplify the competitive dominance of floating-leaved plants over submerged plants? [Dataset]. Zenodo. <https://doi.org/10.5281/zenodo.21964113>.

Xiong F, Li Z, Brosse S et al. 2026 Fishing ban halts seven decades of biodiversity decline in the Yangtze River. *Science* 391(6786), 719-723.

Xiong X, Huang W, Dong Q, Zhang D, Wan S, Gao Z. 2023 Genetic parameter estimates for intermuscular bone trait in grass carp (*Ctenopharyngodon idella*). *Aquaculture* 563, 739011.

Zhen W, Zhang X, Guan B et al. 2018 Stocking of herbivorous fish in eutrophic shallow clear-water lakes to reduce standing height of submerged macrophytes while maintaining their biomass. *Ecol. Eng.* 113, 61–64.

Zheng X, Xu G, Jeppesen E et al. 2026 Water level reduction and fish stock management promote restoration of submerged plants in shallow lakes, while enhancing phytoplankton diversity and stability. *Water. Res.* 126068.

Zhi Y, Cao Y, Sun J, Li W, Jeppesen E. 2018 Indirect effects of extreme precipitation on the growth of *Vallisneria denseserrulata* Makino. *Environ. Exp. Bot.* 153, 229-235.

Zhou Y, Li X, Zhao Y et al. 2016 Divergences in reproductive strategy explain the distribution ranges of *Vallisneria* species in China. *Aquat. Bot.* 132, 41–48.
